# Supplementary material for: ADAM17 Is an Essential Factor for the Infection of Bovine Cells with Pestiviruses
Source: Viruses. 2022 Feb 13;14(2):381. doi: 10.3390/v14020381 (PMC8875743; doi:10.3390/v14020381)
Supplement: Supplementary file 1 [file viruses-14-00381-s001.zip › viruses-1589460-supplementary.pdf]

viruses

MDPI

Supplementary Materials

ADAM17 is an Essential Factor for the Infection of Bovine Cells with Pestiviruses

Marianne Zaruba [1], Hann-Wei Chen [1], Ole Frithjof Pietsch [1], Kati Szakmary-Braendle [1], Angelika Auer [1], Marlene Mötz [1], Kerstin Seitz [1], Stefan Düsterhöft [2], Aspen M. Workman [3], Till Rümenapf [1] and Christiane Riedel [1,*]

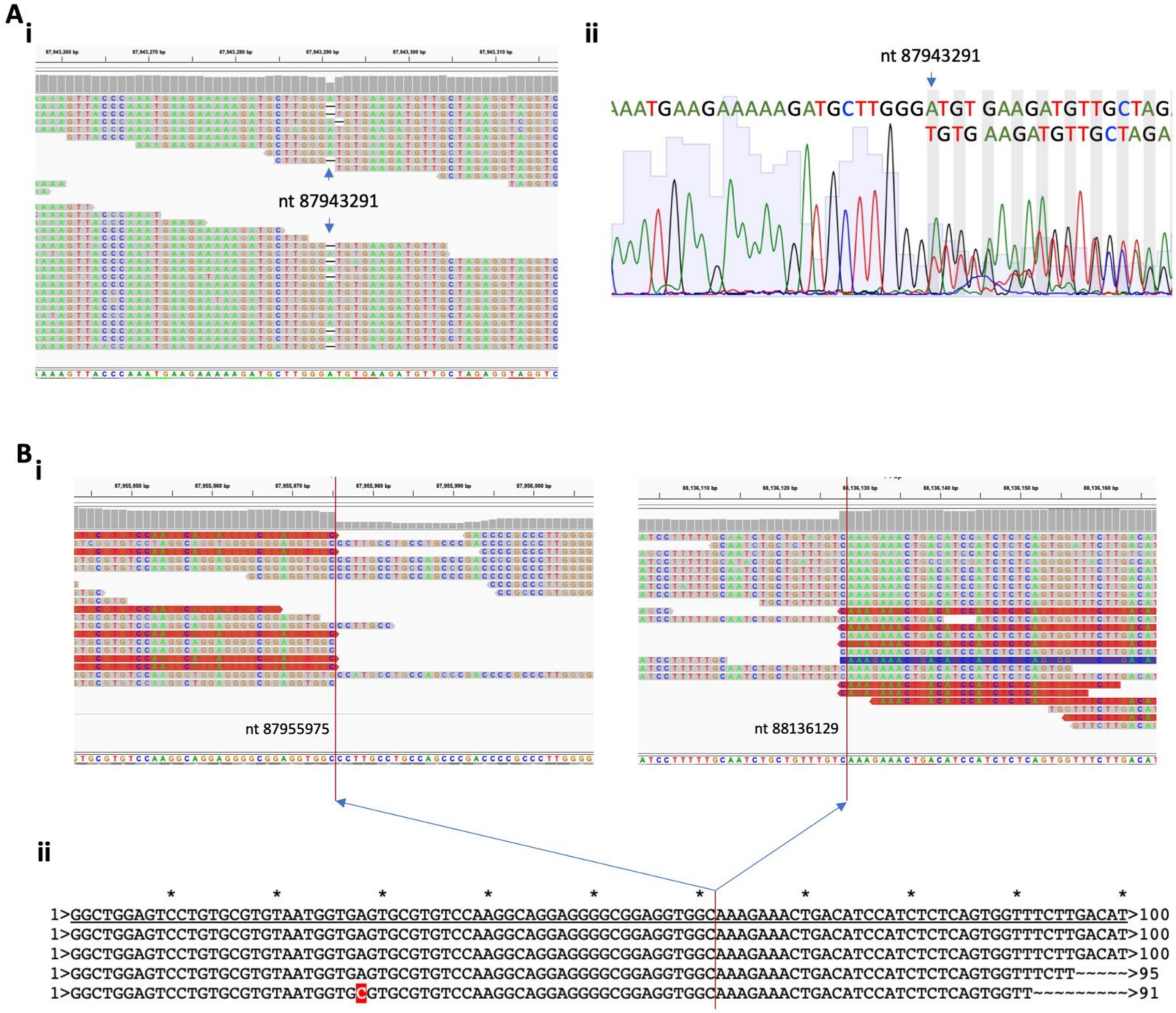

Figure S1. Modifications in the *ADAM17* gene. (**A**) (i) Alignment of the reads in the vicinity of the deletion of the nucleotide at position 87943291 and (ii) sequencing chromatogram of a PCR amplicon of exon 8 of the ADAM17 gene demonstrating the deletion at position 87943291 on one allele. (**B**) (i) Alignments of the reads at the beginning and the end of the large deletion. (ii) Alignment of reads covering the gap caused by the large deletion.

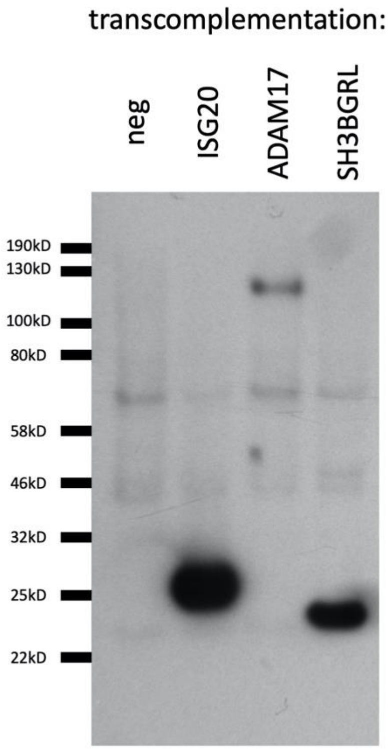

Figure S2. Detection of ADAM17 and SH3BGRL expression in lysates of HEK293 cells upon transcomplementation by Western blot analysis employing an anti-flag monoclonal antibody. Neg = negative control.

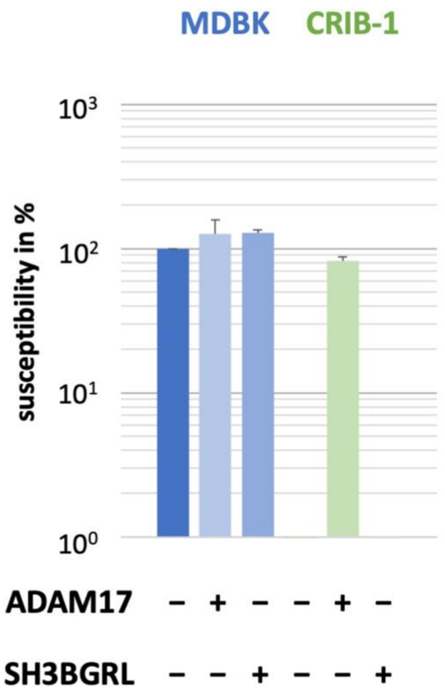

Figure S3. Susceptibility of MDBK and CRIB-1 cells expressing bovine ADAM17 or SH3BGRL to a BVDV clone labeled with a fluorophore at the N-terminus of E2. Shown are the average and the standard deviation of three independent experiments.

Table S1. List of primers and probes employed in this study.

Name	Sequence	Purpose
3′ ADAM17 RT rev	GTCCAGGAAGTCAGCCCGGTATG	reverse transcription of ADAM17 mRNA
5′ ADAM17 for	GACCATGAGGCACCGTGTCCTC	Amplification of ADAM17 cDNA
3′ ADAM17 rev	CGGTATGCGGCTGGGGACTA	
5′ ADAM17 for cloning	CTGCCACCATGAGGCACCGTGTCCTCTTGC	Amplification of ADAM17 cDNA for cloning
3′ ADAM17 rev cloning	GTCTTTGTAATCGCACTCGGTCTCCTTGCTGTC	
5′ ADAM17 rev cloning	CACGGTGCCTCATGGTGGCAGCGCTCTAGAAC	Amplification of vector for integration of ADAM17
3′ ADAM17 for cloning	CAAGGAGACCGAGTGCGATTACAAAGAC-GATGACG	
ADAM17 for qPCR	GACCAGGGAGGGAAGTATGTCATG	Quantification of ADAM17
ADAM17 rev qPCR	CTGGAAACACTCCTGGGACTTGC	
SH3BGRL for qPCR	CCAAGATGGTGATCCGTGTGTA	Quantification of SH3BGRL
SH3BGRL rev qPCR	GGTATCCTGTGGCTGGTCGA	
5′ SH3BGRL for cloning	GCTGCCACCATGGTGATCCGTGTGTATATTGC	Amplification of SH3BGRL cDNA for cloning
3′ SH3BGRL rev cloning	GTCTTTGTAATCTGCTTGCTGCTTTGCCTGAGC	
3′ SH3BGRL for cloning	GCAAAGCAGCAAGCAGATTACAAAGAC-GATGACG	Amplification of vector for integration of SH3BGRL
5′ SH3BGRL rev cloning	CACGGATCACCATGGTGGCAGCGCTCTAGAAC	
actin for	CAGCACAATGAAGATCAAGATCATC	Quantification of beta actin
